# Information technology resource management in radiation oncology^*^

**DOI:** 10.1120/jacmp.v10i4.3116

**Published:** 2009-09-02

**Authors:** R. Alfredo Siochi, Peter Balter, Charles D. Bloch, Harry S. Bushe, Charles S. Mayo, Bruce H. Curran, Wenzheng Feng, George C. Kagadis, Thomas H. Kirby, Robin L. Stern

**Affiliations:** ^1^ Department of Radiation Oncology University of Iowa Hospitals and Clinics Iowa City IA USA; ^2^ UT MD Anderson Cancer Center Houston TX USA; ^3^ Department of Radiation Oncology Washington University Saint Louis MO USA; ^4^ Radiation Oncology Dept. UMass Medical Center Worcester MA USA; ^5^ Department of Radiation Oncology Rhode Island Hospital Providence RI USA; ^6^ Department of Radiation Oncology William Beaumont Hospital Royal Oak MI USA; ^7^ Department of Medical Physics School of Medicine University of Patras Rion Greece; ^8^ Global Physics Solutions/Univ. New Mexico Albuquerque NM USA; ^9^ Department of Radiation Oncology University of California Davis Health System Sacramento CA USA

**Keywords:** Picture Archiving and Communication Systems, information technology, information services

## Abstract

The ever‐increasing data demands in a radiation oncology (RO) clinic require medical physicists to have a clearer understanding of information technology (IT) resource management issues. Clear lines of collaboration and communication among administrators, medical physicists, IT staff, equipment service engineers, and vendors need to be established. In order to develop a better understanding of the clinical needs and responsibilities of these various groups, an overview of the role of IT in RO is provided. This is followed by a list of IT‐related tasks and a resource map. The skill set and knowledge required to implement these tasks are described for the various RO professionals. Finally, various models for assessing one's IT resource needs are described. The exposition of ideas in this white paper is intended to be broad, in order to raise the level of awareness of the RO community; the details behind these concepts will not be given here and are best left to future task group reports.

PACS number: 87.52.Tr, 87.53.St, 87.53.Xd, 87.90.+y

## I. INTRODUCTION

Radiation oncology (RO) is a patient‐centered endeavor and, consequently, the entire clinical workflow and its supporting information technology (IT) infrastructure need oversight from medical physicists and other RO personnel who understand the critical nature of patient data. Patient‐related data are generated when patients are imaged, planned, localized, and treated. Data integrity from one step to the next must be preserved. The data is used often and must be available in real time at the treatment machines. The medical physicist understands the workflow and the data transactions, and must therefore be involved in all IT decisions that affect patient care. This need to be involved in IT decisions is even more critical in the era of intensity‐modulated radiation therapy (IMRT), image‐guided radiation therapy (IGRT), four‐dimensional radiation therapy (4DRT), electronic medical records (EMR), informatics, and the paperless clinic – all of which have resulted in a dramatic increase in the volume of data and supporting computing infrastructure.

These new demands require new models of RO IT resource management. Reliance on the clinic's IT staff alone will not suffice, not only because manpower is limited, but also because a majority of IT personnel do not fully understand the critical needs of the RO IT environment. Careful integration of imaging, planning and treatment equipment into the clinic's network (and its broader implications for the wider hospital network) involves careful planning to design the appropriate network architectures and data storage, backup and retrieval models. Such planning requires collaboration among the medical physicists, equipment service engineers, RO IT staff, and hospital or clinic IT staff. The real‐time nature of treatment data requires a dedicated RO IT professional to be immediately available when problems arise in the middle of a treatment procedure. The resolution of issues can only be achieved with a deeper understanding of the RO‐specific data on the part of the RO IT professional and the medical physicist. Integration with hospital‐wide information systems would be facilitated by the use of resource maps that clearly indicate what RO IT‐related resources are available, how they interact, what is required to manage them, and who is responsible for these various tasks. IT security policies sometimes interfere with equipment service, clinical operations, and patient treatment safety issues; a resolution of such conflicting concerns requires cooperative goalsetting, as well as a clear understanding of the IT needs in RO.

An awareness of these issues by medical physicists will enable RO clinics to take the first steps towards improving their IT processes. An evaluation of a department's IT resources, hospital or clinic IT interfaces and collaborations, as well as RO‐specific IT training for the medical physicists and IT professionals can then be performed with a common vision and understanding of these unique requirements. The AAPM Working Group on Information Technology (WGIT) aims to highlight that vision, using this white paper to raise these broad issues and start the necessary dialogue. In order to do so, this paper provides an overview of IT in RO, a typical list of IT related tasks and resource maps, the skill set and knowledge required for these various tasks, and models for determining a department's IT resource needs.

This paper is intentionally broad and general so as to raise awareness of the issues for a larger audience that consists of all the groups involved: administrators, IT staff, medical physicists, service engineers, and vendors. However, it does not provide in‐depth recommendations that are best left to more focused task groups. For instance, the tables of computer skills are meant to be examples of the knowledge that will be useful for medical physicists, not an excerpt for a job description. A fully explained curriculum will be left to a future task group report. Readers are encouraged to contact the WGIT by sending email to ralfredo-siochi@uiowa.edu if they think that particular issues should be further elucidated in a task group report or in articles that specifically address members of their profession. Since medical physicists and RO departments are ultimately responsible for accurate IT system functionality and patient data integrity, the WGIT begins the dialogue in a medical physics venue.

## II. OVERVIEW OF IT IN RO

**Figure 1:**
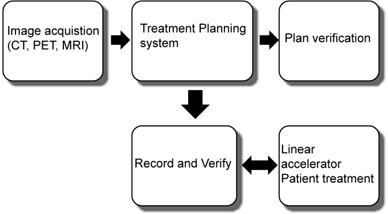
Schematic of a typical radiation therapy process flow.

Radiation Oncology is very dependent on the computer control of sophisticated electronic equipment, information transfer over hard connections as well as networks, and data storage and retrieval. RO is one of the most IT‐demanding areas of the hospital. Computer programs such as Treatment Planning Systems (TPS), Treatment Management Systems (TMS), Treatment Delivery Systems (TDS), RO specific EMR (RO EMR), and image viewing systems (e.g. Picture Archiving and Communication Systems (PACS)) are used on a routine basis. The term “Record and Verify (R&V)” has been used to describe systems that receive data from the TPS and interact with the TDS to deliver radiation. All of these systems, controls, and networks must operate smoothly if the RO process is to be safe and efficient. Figure 1 illustrates the processes involved. Each process can transfer information through any combination of hard wiring, intranet, local area networks, and the Internet.

Once the decision has been made to treat a particular patient, appropriate images are acquired. These images may be taken by CT, MRI, PET or other modalities. The requirements and protocols for these images are often quite different from those taken for diagnostic purposes. Because image manipulation is not usually done through the imaging device software, the images are sent to the treatment planning system (TPS). This transfer is most often accomplished via a local area network, but could also be through some transportable media such as DVDs. The images are typically transferred using Digital Imaging and Communications in Medicine (DICOM) formats and transfer protocols.^(1)^

The TPS is the heart of the RO process. It consists of hardware and software specifically designed for RO. Images are uploaded and prepared for the treatment planning process. Physicians delineate areas to be treated on CT images, for example, and generate a prescription that includes a set of treatment parameters. These parameters include the amount of radiation (absorbed dose) to be delivered to the target, as well as limitations on the maximum amount of radiation to be delivered to uninvolved areas in the patient. A dosimetrist or physicist then works with the TPS to achieve the physician's goals. In some cases, plan information is sent to remote workstations for review and approval by other medical specialists. The resultant treatment plan is a combination of images, contours, calculated radiation dose distributions, and parameters used to control the treatment machine. These data, frequently consisting of hundreds of megabytes per patient, are then stored on the local hard drive of the TPS. Older data are eventually archived and removed.

In order to execute the treatment plan, the parameters that control the linear accelerator (linac) are then transferred from the TPS to a database accessible by the RO EMR. Before final approval of the treatment, a physicist reviews the plan. In the case of complex plans such as IMRT, the treatment is delivered to a test phantom and measurements of the radiation distribution are compared to calculated values. This involves yet another transfer of data among systems.

Once the plan has been reviewed and approved, the plan in the RO EMR is electronically signed and released for treatment. This EMR may not be from the same manufacturer as either the TPS or linac, so communication and networking compatibility issues require careful consideration. The linac itself consists of many computer‐controlled subsystems that may or may not be affected by network issues.

The RO EMR controls, verifies, and records all aspects of each individual treatment. Patients are typically treated once a day, five days a week, for a period of two to seven weeks, depending on the type of cancer. Each time the patient is treated, the linac requests the treatment parameters from the EMR, positions the beam‐defining devices, informs the EMR database of its positions, and waits for the EMR to verify that the positions are within tolerance. Once the linac receives the approval, it delivers the radiation and sends the delivered treatment information to the EMR so that it can record the dose and treatment parameters that were used to treat the patient. This process of downloading, verifying, treating, and recording is repeated for every single treatment field. For simple cases, patients may be treated by anywhere from 1 to 4 fields, while for IMRT, as many as 100 fields may be used. It is important that the network infrastructure efficiently handle the transfer of these large amounts of data ‐ otherwise, patient treatment could be either delayed or compromised. The interaction between the linac and the EMR also requires real‐time support; the patient must be treated as quickly as possible in order to minimize the chance that the patient has moved from the target position.

Those who interact with the RO EMR must recognize that incorrect data or the incorrect operation, use, installation, and maintenance of this EMR can have disastrous consequences, potentially delivering fatal doses of radiation in a very short time. The RO EMR is very different from the typical concept of an EMR in that the RO EMR supplies critical information for the control of radiation treatment machines. Nor is it the Record and Verify system of the early days of computer‐controlled treatment deliveries, because it contains much more information than what is required to monitor a treatment. Appropriate permission levels must be given to users so that only those who fully understand the linac control data in the EMR are allowed access to it.

There are many hardware, software, and networking issues in RO. Integration of all of these systems is essential for proper and efficient treatment of the patients. This integration may be provided by a single vendor, multiple vendors, or may have to be performed by local personnel. Each process generally has its own hardware and software. It is not uncommon to find Windows, UNIX and Linux operating systems, which all need to communicate. Changes to hardware, software, and network infrastructure all need to be tested to preserve proper integration. It is therefore essential that the RO personnel have trained, cooperative and accessible IT support. Conversely, the IT personnel need to have the support and cooperation of the RO department.

## III. IT‐RELATED TASKS

As the RO clinic guides a patient from imaging to treatment, a large number of IT related activities occur in support of patient care. Some activities directly affect a patient and may be thought of as “real‐time” clinical processes. Problems that arise in these processes require immediate attention. Other activities improve the ability of the clinic to provide cutting‐edge treatments; these include research, teaching, process improvement, and documentation. The role of IT in these activities is sometimes overlooked, and it is important for RO management decision makers to understand the impact of these specialized IT tasks on the operation of the clinic. This section attempts to portray the breadth of such tasks. The descriptions that follow are based on conventional external beam linac treatment delivery systems. Other external beam treatment delivery systems are similar in scale to linear accelerators with respect to IT resources, but have their own specialized IT resource requirements, and will not be described here. Nor will this white paper deal with the IT requirements needed for brachytherapy systems, such as those for High‐dose Rate (HDR) treatments and permanent prostate implants. Finally, in some settings, these IT tasks might be performed by Biomedical department personnel or by other individuals, and they should be included in discussions where their roles overlap with the descriptions here.

### A. Treatment

In order for treatments to be delivered in most radiotherapy clinics today, parameters necessary for the control of medical devices are electronically parsed out from a TMS database to the computers that control each device. Depending on the capabilities and vendor selection for a given treatment unit, it is not uncommon for a single console area to have at least 5 computers (typically PCs) and potentially as many as a dozen or more.

#### A.1 Site planning of the treatment area

The planning stage includes specification of ergonomic design (e.g. light levels for optimal monitor viewing), network jack locations, IP space (dynamic and/or static), firewall configuration, cooling requirements, security access, and virus protection. It also involves identifying exceptions to IT policies due to computer configurations that are required by the vendor for FDA compliance and/or remote support. (For example, some antivirus software can interfere with applications on vendor systems that are used to deliver treatments.) This planning stage involves cooperation between RO, hospital or clinic IT, and the vendor's staff, with a medical physicist serving as a project manager and point person for all IT related assessments.

#### A.2 Installation

The installation of equipment that is IT in nature (RO EMR), or that has an IT component (e.g., imaging systems, MLC), is performed by the respective equipment vendors. The coordination of the overall project should always involve a medical physicist and is typically led by the medical physicist and/or onsite engineering staff. Typically, the vendor's computers will be preloaded with the FDA‐approved operating system and software configuration, but one or more PCs with the standard hospital configuration may also be present in the console area. The post‐installation acceptance testing and commissioning of the various components should also include the IT‐related aspects, such as communications and secure access.

#### A.3 Support

The initial response to IT‐related problems at the time of treatment must be of high priority and prompt (i.e. less than 3 minutes), and also be provided by personnel that can resolve the situation quickly. Typically, and optimally, the initial response comes from the medical physicists who then should determine if they can solve the problem (e.g. database or DICOM server reboot), or if the issue is best solved by hospital or clinic IT staff (e.g. network connectivity, domain/rights issues), vendor or in‐house engineering staff (e.g. MLC communications), vendor applications support (e.g. application error messages, DICOM configuration/server issues), or other RO staff (problems with input data). The response times required of all involved parties to resolve the problem completely should be clinically appropriate as determined by the medical physicist.

#### A.4 Business continuity

In the event of IT‐related system failures (e.g. network is down, EMR database is off‐line), contingency plans must take effect. While other tasks can be affected by a system failure, the most critical ones are related to treatment delivery. *Ad‐hoc* responses are not appropriate, and plans must be developed to deal with the most likely failure modes. The solutions may require the purchase of additional hardware and software (e.g. mirrored backup systems for fast switch‐over from the primary RO EMR database to its backup) and should be tested to ensure that the down time is kept below a clinically appropriate maximum. In many cases, the plans will require IT support (e.g. network problems) and IT staff should be identified for RO‐specific training to handle these contingencies. The medical physicist should collaborate with IT to develop these plans.

### B. Pretreatment and post‐treatment

RO information systems today provide not only R&V capabilities but also a central database for most, if not all, of the data needed for treatment and documentation. The data collection begins as soon as the patient enters the department for a consult with the radiation oncologist. Because of the dependence of treatment planning and treatment on imaging, the IT resource requirements increase rapidly as the patient progresses through their course(s) of treatment.

#### B.1 Hospital information systems

RO departments within a hospital system depend on the hospital‐wide information system (HIS). Patients are typically registered in the HIS, which serves as a source of patient demographic, billing, and insurance information. It is quite common to exchange data between the HIS and the RO information system. The HIS also provides clinical, laboratory, and radiology information. Support for the HIS is typically provided by the HIS support staff. If there is communication between the hospital and departmental system for registration, billing, and transcription, then the interface (usually encoded using the Health Level 7 (HL7) standard) between the systems requires RO staff (often the physicist) to mediate between the hospital and the RO IT vendor.

#### B.2 CT simulation

For departments with dedicated CT scanners, image sets are electronically transferred (typically via DICOM) to a CT simulation workstation where the isocenter and setup field coordinates are determined. These coordinates are then transferred to the CT laser marking system for marking the patient's skin. Since this process (from scan to marking) is done with the patient in the treatment position on the CT couch, it is necessary to have IT support readily available.

#### B.3 Treatment planning systems

Treatment planning workstations are typically vendor‐configured with operating system and application software. The system is usually not compliant with the hospital or clinic IT specifications that qualify a computer to receive support. The medical physics staff provide support in these cases, in close association with the vendor and IT staff, since the workstations are usually on the hospital or clinic network.

#### B.4 Other radiation oncology information system software

As noted above, RO information systems provide applications to support scheduling, billing, paperless charting, image storage, retrieval‐and‐review, and more. Support for these applications is typically required of the medical physics staff or a dedicated RO IT staff member (if available), who will work closely with the vendor and medical physicist.

#### B.5 Data transfer to other institutions

Preparation of treatment plan data for export to protocol groups (e.g. RTOG, ITC, QARC) is usually performed by a medical physicist or dosimetrist. However, security, access, and HIPAA requirements often necessitate IT support. The medical physicist, IT staff, and the protocol group need to collaborate to ensure that the data reaches the protocol group on schedule, in the right format, and in compliance with all the relevant regulations. Cooperation from IT staff is crucial, as many IT policies tend to impede the transmission of such data. Information technology administration should be educated about the nature of this data and the importance of contributing to the body of clinical knowledge that will improve patient care, and so be required to find a solution that will allow the transmission.

#### B.6 HIPAA

While communications with outside institutions may be the primary reason for HIPAA‐related security measures, HIPAA activities are not limited to such data transfers. Datasets used by researchers within RO must always be anonymous, files that track patients with datasets must be secured, and security policies for computers and various data storage systems that contain patient data need to be developed. As RO starts to realize the possibilities of informatics research, mechanisms for ensuring HIPAA compliance will have to adapt to the increasing demands for patient data.

### C. Maintenance

#### C.1 Backup and archiving

Scheduled backups (typically daily) are necessary for recovery from storage or server system failures. Tape backup using the Digital Linear Tape (DLT) technology is quite common for database and image server backup. This requires that tapes be changed and stored periodically. It also requires monitoring and testing of the backup system not only for ensuring proper performance but also for providing uninterrupted access to the real‐time treatment databases. Archiving patient data to storage media (optical or tape) is often used to free up space and improve performance of the database system as well as other systems that access the data and images. Sufficiently remote off‐site backup must be an integral part of this process to avoid data loss during catastrophic situations (e.g. Hurricane Katrina's effects on New Orleans, LA).

#### C.2 Database maintenance

The database software used for most RO information systems has features so that errors, messages, and warnings pertaining to the “health” of the system can be monitored. This is often done by the use of log files that are constantly updated. These files should be periodically reviewed to ascertain if any preemptive action can be taken in order to avoid impending downtime. Also, most systems have a “dump” feature where the data is frequently (often hourly) written to a separate file, so that the database can be restored to a recent state in case of failure. This process should be periodically monitored, under a medical physicist's supervision, to make sure that it is working properly.

### D. Miscellaneous

#### D.1 Wiki

A wiki is a collaborative Web environment allowing those that access it to contribute their knowledge and/or modify the content by using simplified markup language.^(2)^ There is a wide variety of available wikis that have been implemented in order to foster collaboration over the Internet (or the intranet). Wikis have been used with success for interdepartmental collaboration^(^ ^3^ ^–^ ^6^ ^)^ and departmental procedures. In some clinics, they are used to communicate the status of treatment equipment, special treatment procedures, clinical processes, and planning protocols. During the implementation of new technology or procedures, it becomes a critical part of staff training. Because its content can change frequently, it needs to be managed by a wiki administrator.

#### D.2 Websites

Many medical physics departments have their own websites, with information for radiology, radiation therapy, and medical physics trainees, as well as information for research protocols and collaborations. Likewise, individual medical physics associations, such as the American Association of Physicists in Medicine (AAPM)^(7)^ have an established archive of information for their members. IT staff should allow access to these websites as they provide a resource that is crucial to professional development and improvements in patient care. They should also support internal websites with proper documentation to ensure these sites stay functional, even if they must be managed by a different administrator.

#### D.3 Satellite facilities and inter‐clinic IT integration

Many facilities operate widely dispersed satellite clinics, and remote support is often the only way to solve clinical issues in a timely manner. In addition, scientists and departments may wish to collaborate with others who are at different geographical locations. For these situations, Virtual Network Computing (VNC) and Virtual Private Networks (VPN) have provided the means for secure and safe communications, in which data is transmitted without error, in a timely manner, and only to the people who are authorized to receive it. VNC is a system that uses the RFB protocol for remote control of a distant computer.^(8)^ It is a platform‐independent system within which a client can use both the keyboard and the mouse of a server. VPN is a computer network in which the linking between specific nodes is performed by virtual circuits or open connections in networks like the Internet, instead of using physical wiring technology.^(9)^ VPNs can be used to separate the traffic of specific user communities over an underlying network with strong security features. With their application, designated users can interact and function without problems as if they were using the same computer laboratory. Encryption protocols are important to preserve data security, while firewalls are specific systems that are programmed to filter data between domains of different security specifications. With their implementation, system administrators (sysadmins) can be assured that designated user groups have specific privileges and access to specific data and services.

These technologies can not be applied indiscriminately using standard IT policies. Input by the medical physicists at the various clinics is required to ensure that they can access all the data they need to do their work. Real‐time clinical (patient) support must be carefully considered when choosing the technology to be implemented.

#### D.4 General computing administration

Computing support is provided by sysadmins. They have a variety of duties that may differ in accordance with the organization they serve. Sysadmins are usually responsible for installing, supporting, and managing computer systems. They have to provide technical support, develop websites, perform routine maintenance, and manage security issues, access levels, and installation of clinical software, PACS, and Radiotherapy PACS (RT‐PACS). General technical support (e.g. MS office installation) is usually provided by technicians assisting the whole IT procedure. In RO departments, information technology issues related to PACS, RT‐PACS, DICOM, HIS, and Radiology Information Systems (RIS) are usually addressed by the responsible medical physicist.^(10)^ Medical physicists have developed expertise in the field and are responsible for management decisions and the proper dissemination of information needed in the healthcare enterprise.^(11)^ A medical physicist, or a second sysadmin, should be given administrative rights or passwords to act as a backup for the primary sysadmin. Hospital sysadmins should never make changes to RO IT systems without consultation and agreement with the medical physicist.

#### D.5 Network infrastructure

The most common networks that an end user encounters are the LANs (Local Area Network). As implied by the name, LANs usually reside in a single building, in a complex of buildings or on a campus up to a few kilometers in size (less than 10 km).^(12)^ They are mostly used to share resources (such as printers, files, internet connections, etc.) and exchange data among components of the local IT infrastructure (personal computers, workstations and servers). The other main attribute that discriminates LANs from other network types is the in‐advance knowledge of all main network characteristics (size, physical layer technologies and topology). Both the small size and the in‐advance knowledge of the main network characteristics make LANs simpler to design and manage when compared with other network types. LANs are common in Radiology and RO departments, and must have sufficient bandwidth for specialized services. In some cases 100 Mbps is insufficient for quality data transfer. These networks must also have high availability support mechanisms in order to archive and backup digital data that are critical to the patient's health care (diagnosis, therapy, follow‐up), and must be properly developed in order to support the plethora of different client and/or server systems throughout the hospital. Real‐time treatment support requires an extremely high uptime for these networks, so the network architecture must be designed with alternative routes in case of failure. Medical physicists should be consulted when the systems are being designed.

Wireless networks have additional considerations. Current technology limits the transmission speed to approximately 140 Mbps, so these may not be suitable for some applications. Their operating frequencies (2.4 to 5 GHz) may also interfere with those of other RF systems, or the other systems (e.g. linac at 3 GHz) may cause problems for these networks. Finally, security policies for wired and wireless networks may be different and the impact of this on clinical systems must be discussed with the medical physicist.

#### D.6 Vendor support

To provide real‐time support, vendors may prefer to use remote desktop applications (e.g. automated log file transfer). These allow the vendor to use a secure Internet connection to view and interact with the problematic computer. It has the advantage of providing even remote clinics with immediate access to highly skilled vendor support staff. Coordinated effort by the medical physicist, the vendor, and the IT staff is needed to configure and assure compliance of the remote access application with the clinic's security policies. The IT staff will likely need to make modifications to firewalls, proxy settings, host intrusion prevention software, antivirus software, and/or other network configuration parameters, in order to enable the connections.

### E. Research

Commercial TMSs (e.g. IMPAC, Aria) and TPSs increasingly utilize databases to store a wide range of valuable data about patients and their treatments. This sets the stage for enabling clinics to efficiently carry out retrospective examinations of patient data to search for correlations that may be used to guide future clinical decisions. This is an extremely valuable tool for the emerging demands for evidence‐based medical practice, research and quality assurance. To mine this data, clinics require individuals with skills in database design, queries, and intermediate reporting tools. Database design requires understanding of both the underlying software technology of the database, and of the nature and use of the information to be stored in the database. It also depends on the type of information one desires to pull from the database through queries. Manually creating SQL queries to extract information from many tables, subject to a broad set of constraints, can be difficult. To simplify this effort, intermediate reporting tools are used, but require an understanding of relational database concepts in order to be used effectively. These tools also have the capability of producing user‐generated customized reports of the information returned from one or several queries.

To effectively pursue research, the knowledge domain of medical physics must be combined with the skill sets for database handling. This will require collaboration between IT professionals and the medical physicist. Information from the R&V database may need to be combined with other databases used for clinical trials. Such efforts often require the use of cutting‐edge IT technology, and custom software is then required to support these endeavors.^(13)^ Commercially‐available solutions may not be sufficiently integrated, and programmers may need to tailor them to make them clinically functional.

### F. Teaching

Audiovisual support is closely tied to IT support, since many lectures are given through PowerPoint slides. The use of computers is also quite prevalent in many new technologies that emerge from physics education research.^(14)^ Numerous educational resources are available on the Internet. Vendor training often happens through WebEx as well, or self‐paced Internet courses that require various videoplayers. To take advantage of improvements in education, teaching institutions need support from IT staff for the implementation and maintenance of these emerging technologies.

## IV. CLASSIFICATION OF RT COMPUTER SYSTEMS AND RELATED NETWORK TOPOLOGY

The variety of IT‐related tasks usually involves a complex mixture of various computer systems with different security and networking requirements. This presents a challenge when defining appropriate support and control of computing resources in radiation therapy. Generally all computing systems in radiation therapy need support or oversight via cooperative efforts between medical physicists and hospital and department IT staff. However, the roles in the collaboration and the levels of responsibility depend on the nature of the system in question.

**Figure 2:**
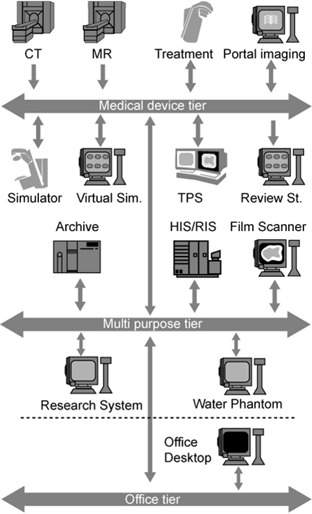
The three tiers of computing resources and their connections.

To address this latter point, we categorized various systems into three classes: medical device, office, and multi‐purpose tiers. A general description of these tiers follows, with examples of systems for each. In addition, network connectivity is related to tier level, and so a generic topology map is included (Fig. 2). Table 1 provides a summary of the roles and responsibilities classified by tier, while Table 2 summarizes the characteristics of the various tiers. The descriptions illustrate how these systems should be considered in an organization's network architecture. Alternative schemes that follow the principles outlined here may be possible.

**Table 1:** Roles classified by tiers.

*Tier*	*Staff*	*Roles*
Medical Device	Medical Physicist (P)	Initial system tests to verify system is safe and functioning according to specifications. Periodic QA tests to verify system is still in specifications. Internal policies and procedures regarding system use.
	IT (S)	Power and environment for hardware. Network connectivity to meet requirements specified by vendor and to meet needs determined by medical physicist. System security in accordance with specifications provided by vendor and medical physicist. Patient data backup and archiving.
	Vendor (S)	Specifies system configuration; provides software and upgrades, provides technical support.
Office	IT (P)	Power and environment for hardware. Software and support. Network connectivity to clinical needs. System security in accordance with hospital and / or clinic policies.
	Medical Physicist (S)	Provides IT with a list of system requirements to meet clinical needs.
	Vendor (S)	Provides initial system with configuration as specified by IT. Provides software, upgrades, and technical support as requested by IT.
Multipurpose	Medical Physicist (P)	See the description for the medical device tier.
	IT (S)	In addition to the items described in the medical device tier: Additional software and support as required by medical physics.
	Vendor (S)	See the description for the medical device tier.

P=Primary and S=Secondary responsibility.

**Table 2:** Characteristics of the three IT system tiers.

*Category \ Tier*	*Medical Device*	*Office*	*Multipurpose*
Patient Interaction	Direct	None	Indirect
Primary Responsibility	Medical physicist	IT	Medical physicist
Vendor Support	Often	Rare	Often
IT Support	Must go through medical physicist	Per IT Policy	As requested by physicist
Connectivity (clinic)	Isolated from other network traffic, high bandwidth	Limited to non‐clinical tier systems	Full
Connectivity (outside)	Virtually no direct internet access (exception: remote vendor support)	Full	Full

### A. Medical device tier

Computer systems in this group are entirely clinical. They generally have specialized or limited functions. They have little (e.g. remote service) or no need for Internet connections outside the hospital system, and special attention must be given to security and access of such devices. They may have high bandwidth requirements within the clinic or the hospital. Because they directly affect patient care, these systems require the most oversight by medical physics. Their configuration and upgrades will be done primarily by the vendor or the medical physics staff, but may also require assistance from hospital or clinic IT (e.g. data backup and archiving, network connectivity).

Examples of computers in this tier include: linear accelerator control computers (“console”), MLC control computers, portal imaging computers, CT simulator computer systems, and TPSs. In the past, these computers were often not Windows‐based and sometimes had no network connectivity. As standalone proprietary systems, they were primarily controlled by medical physicists, with little or no IT support (for data backup, for example). Today, however, all computers are likely to be networked and can be Windows‐based PC platforms, too. Because such systems are quite similar to office desktop computers, the delineation of support roles can be misunderstood. Medical physicists must work with IT to identify the computers in this class and establish specific rules governing responsibility for these systems.

### B. Office tier

At the other end of the spectrum are common desktop computers, typically deployed by IT. These are multifunction computers that usually have little clinical function. In particular, they have no direct impact on patient care. They may include software for accessing clinical systems (e.g. patient scheduling, billing, electronic chart review), but they are not specialized for those tasks.

Examples of computers in this tier are the desktop computers of most personnel in RO. These computers will have relatively unlimited access to the Internet. These systems have considerable demand for network bandwidth, in part because there are more of them than those found in any of the other tiers, and they may also be transferring significant amounts of data over the Internet. However, most of the network traffic from the computers in this tier is not critical to real‐time patient treatment activities, and therefore should be routed so it does not interfere with network traffic among the medical device tier computers. Computers in the office tier should be the primary responsibility of IT, with support coming from medical physics for issues with clinical clients (e.g. R&V software client).

### C. Multi‐purpose tier

Computers in this tier are intermediate between the completely clinical systems and generic office systems, and operate in a “grey area” of multipurpose use. These computers, while not entirely clinical, need access and connectivity to clinical systems. However, they may also need many of the functions (Internet connectivity, spreadsheet software, etc.) of the non‐clinical desktop computers. Medical physicists will need to work most closely with IT to manage these systems.

Computers in this class include not only the physicist's office desktop system but also computers that control film and chamber dosimetry systems. A water phantom scanning system controls the water phantom and acquires beam data. It needs connectivity to the clinical systems, for example, to transfer commissioning data to the TPS. However, it may also require generic functions and connectivity outside the clinic. It might need to have office spreadsheet software for analysis of beam data, or it may need to be able to generate figures from that data and electronically exchange information with journals or outside collaborators. Similarly a medical physicist's desktop computer generally needs Internet connectivity for email and similar communications operations, but may also need access to TPSs to do further analysis on clinical data. Because of the dual nature of such computers, IT must work closely with the medical physicists to ensure that these systems can meet all of their needs.

### D. Interaction between tiers

The medical physicist needs to be aware that various methods for handling network traffic can produce undesirable interactions among the tiers. For example, in a connection to a remote site, the RO clinic will be allocated some portion of the bandwidth that is shared among all of the site's clinics with the hospital. If IT is not heedful of real‐time connection needs for treatment and IGRT, poor choices in allocation may allow heavy usage in other tiers to degrade the performance in the medical device tier. Similarly, changes in switch and router configurations intended to solve issues for other hospital departments can have sudden effects upon the medical device tier. Therefore, it is important that the medical physicist have access to IT for continued dialog regarding the impact of networking choices among the tiers.

Security across tiers must also be considered when IT policies are developed. For example, research data from medical device and multipurpose tiers are often transferred to office tier computers using flash drives or external hard drives. Protected Health Information (PHI) security and virus‐infected mobile devices can be an issue.

## V. REQUIRED SKILL SET AND KNOWLEDGE

The ability to perform the many IT related tasks described so far depends greatly on the training of the IT professionals. There should be a certain overlap of knowledge that allows them to communicate effectively with physicians, medical physicists, dosimetrists, and therapists. Likewise, since medical physicists have the best understanding of the overall clinical process, they should be familiar with IT concepts and have a solid set of IT skills.

### A. IT and medical physics responsibilities

The delivery of radiation therapy treatments is a complicated endeavor involving the hand‐off of information among systems that span many work groups and different types of equipment from different vendors. This transfer of data is a potential source of concern, with opportunities for both human and system errors compromising the eventual quality of the treatment. The medical physicist is uniquely qualified to understand and implement this overall system. These systems run on physical and virtual networks and computers often maintained by hospital information technology staff. Portions of these systems are composed of software applications that run on desktop computers that are also used for word processing and email. Other portions of these systems run on dedicated equipment control computers that often use common desktop operating systems. Both of these situations blur the lines between personal computers and medical devices.

The safe and effective delivery of radiotherapy can only be facilitated by the proper interaction between medical physicists and institutional IT personnel. In particular, the IT support staff should know what hardware and software they should be supporting and when the equipment engineer or the medical physicist needs to be called. Likewise, the medical physicist must know when the problem requires IT support, and should have a specific person or small group within the IT department assigned to assist in the deployment and maintenance of new or existing radiation oncology systems. This interaction requires the assigned IT support staff to be on site rather than being part of the support that is outsourced to remote private companies.

Two concepts in IT are the “project manager” and the “application owner”; these are the persons who are responsible for leading teams to implement and maintain new systems in the complicated institutional IT infrastructure. The project manager is responsible for organizing a project and its associated teams (e.g. facilities, networking, servers, and software). The application owner is the person who makes all final decisions in the management of a system even though they often do not provide the application support. The project manager should report to a medical physicist for the implementation or upgrade of any direct patient care system in RO. The medical physicist should be the application owner for all medical device tier systems.

### B. Dedicated RO IT professional's training requirements

An IT professional who is dedicated to the RO department should hold a substantially greater skill set than a typical IT professional. This individual should have some of the domain knowledge of the medical physicist and the service engineer and understand the nature of the IT infrastructure in RO. This would enable the dedicated IT individual to collaborate with other RO professionals and play a consulting role in equipment selection, installation, and acceptance.

While such professionals may be called upon to perform general IT tasks, it should be understood that their top priority is to support the clinic. Unfortunately, there are no specific training programs or materials for dedicated RO IT professionals at this moment; most of the training is actually obtained through vendors, on‐the‐job, and by self‐directed study. Tables 3 and 4 provide a list of skills required for the RO IT professional.

**Table 3:** A partial list of generic computer skills, which could be acquired through traditional IT training.

*Category*	*Concept*	*Example Tasks*
Hardware	Computer hardware components, architecture, type, design	Trouble shoot and replace broken parts, upgrade, install and configure UPS, KVM switch, tape libraries or MO/DVD jukeboxes, S‐ATA, SCSI (ID, connector, cable, terminator), RAID
Desktop Software	Operating system (including Windows, Linux, Mac OSX, Solaris, Irix, HP‐UX), firewall, antivirus, spyware, performance monitor, office software support	Operating system setup, driver configuration, firewall rules
Database	Relational model and object‐oriented, Architecture, Indexing, Transactions, concurrency control and database recovery, data mining	Track database growth, performance tune‐up, backup/restore, manual dump, user add/remove, privilege assignment
Productivity Software	Citrix configuration and setup, create custom crystal report, dedicated backup/restore software, VMWare, Ghost	Manual/automatic backup/restore with Veritas BackupExec
Network	Network topology, protocol, TCP/IP (address, DNS, WINS, GW, mask), client/server, devices, bandwidth, Autonegotiation / Autosense, half duplex and full duplex, VPN, SSH/SFTP, X11 forwarding, X Windows client and server, XDMCP, FileServer/NAS (NFS, SMB/CIFS), VNC, logmein	Network setup workstation/servers for TPS/R&V/Linac, router/bridge setup, DHCP binding, network connections troubleshooting
Web	Intranet/Internet web server	Setup and update the website, web design and content update
Research	Research programming	SQL programming, batch/script programming, C/C++ programming with GUI

**Table 4:** A partial list of RO specific skills (most are vendor specific).

*Category*	*Concept*	*Example Tasks*
TPS	TPS specific, data format, coordinate definition, remote access, MU second check software, segmentation and contour software	Data format conversion and transfer between beam scanning software and TPS, model backup, modeled data export, regular cleanup of non‐used data file, backup and restore customer configuration, script creation and modification
R&V	HL7, interface, different format conversion	Integration with HIS, emergency file transfer with faulty network, troubleshooting mismatching
Linac	Console	Connection with R&V, cooperation with localization and couch, backup data
Simulation	CT Sim/MRI/PET, external laser, coordinate translation	Connection configuration, coordinate and structure set matching
Localization	Daily MVCT or KVCT, ultrasound, radiofrequency, optical camera, radiocam, gold seed	Image transferring and archiving
Verification	EPID	Manage image database
Dosimetry	Film scanner, TLD/OSL reader, diode, MOSFET	Upgrade and clean the space
QA	Daily/monthly/annual QA software like Argus	Trend analysis
Scheduling	Patient scheduling and tracking	Real‐time tracking of patient location
DICOM	DICOM/DICOM‐RT, SCU/SCP roles, information object definitions, attributes, data dictionary, service classes, service object pair, association handling, AE title, UID, conformance statement	Setup DICOM node by ip:port AE title, troubleshoot association handling mismatches, (commit, compression, endianness)
PACS	PACS, HIPAA privacy and security regulations, archive, backup, monitor QA, film camera QA	User creation and privilege assignment, query/retrieve, auto‐send, customize LUT

### C. Medical physicist's traditional roles and training in IT

Traditionally, most computer‐related tasks, including system configuration and maintenance, software installation and configuration, clinical commissioning of software, and programming, were the responsibility of the medical physicist. Since very few RO departments had staff specifically trained in computer technology, the medical physicist was usually the person with the strongest computer background. Furthermore, the physicist was the person clinically responsible for many of the software systems such as treatment planning and record‐and‐verify. Therefore, the responsibilities for these systems naturally defaulted to the physicist.

Medical physicists often had little or no formal training in computer technology or networking. What knowledge they did have usually came from research work in graduate school and on‐the‐job training. Even though such skills were known to be an important part of the physicist's job, they were not acknowledged in educational and training recommendations. The guidelines for medical physicist training and responsibilities published by the AAPM in the 1990s and even into the 2000s^(^ ^15^ ^–^ ^19^ ^)^ either do not mention computers at all or simply mention them as an area of knowledge with no specifics given. Only the guideline for academic medical physics graduate program recommendations published in 2002^(20)^ gives any details of what kind of computer‐related skills are needed.

On‐the‐job training was usually sufficient when computer systems were simpler; there was little or no networking within the RO department or the hospital, and departments were not nearly as computer‐dependent as they are now. However, it is a very different world now. Specialized knowledge is required beyond that of an educated layman to adequately set up and administer all the systems and networks needed in a modern department. In addition, IT is changing so fast that a non‐IT person has difficulty devoting enough time to stay current. AAPM Continuing Education (CE) courses, along with updated guidelines for the training of medical physicists, may be needed to address these issues.

### D. Interaction between IT staff and medical physicists

Most hospitals have an extensive system for IT support that often includes separate groups for networking infrastructure, desktop support, and server support. The integration of a new system into this environment often requires interaction with several of these groups. Medical physicists should have adequate knowledge and control of the systems they support and be able to interact with each group in a way that ensures the integrity of the radiation treatment system is maintained. The physicist should be the application owner for medical device tier systems such as the linear accelerators, CT scanners, TPSs, and other devices that they commission and maintain. Whenever possible, the physicist should be involved with the initial design of the network architecture and should be informed well in advance of any changes. The physicist must also maintain administrator rights on these systems and only share these privileges with a small number of IT professionals who have received proper training. These systems should not be grouped into generic management policies intended for general use PCs.

For non‐hospital‐based clinics, the physicist needs to be provided with adequate support services and infrastructure to install and maintain their systems. In contrast to the hospital‐based systems, the physicist may have the responsibility for establishing and maintaining the network, the print and fileservers, and the medical systems. In this situation, the physicist needs adequate hardware and personnel budget to ensure the systems are adequately maintained. This may be a more expensive per unit treatment than in a hospital‐based environment since the basic infrastructure for support and backup will be established without the economy of scale that comes from sharing with other departments.

### E. Linac engineer IT training

The current generation of medical devices, including linear accelerators and CT scanners, often use general purpose computers running commercially available operating systems as front ends and even as control devices. Many of these systems employ several computers on a dedicated local area network (LAN) with one or more connections to the hospital's wide area network (WAN). This presents new challenges for installing and maintaining the equipment.

The hardware infrastructure is often commercially available Ethernet for most connections running on Category 5 or 6 cables. Such cables achieve a high signal‐to‐noise ratio through the use of balanced lines (twisted pairs), reduce the effects of interference, and meet the standards defined by the Telecommunications Industry Association. Most devices employ switches to interconnect the various devices that make up that system. These switches often connect to the hospital WAN through a configurable hardware firewall. The network cabling, and the configuration of the switches and the firewall are often left to the field engineer. In addition, more exotic networking such as optical connections for high‐speed, point‐to‐point links is also employed and must be maintained.

Various networking protocols are used to share information between the subsystems of the machines and with other devices in the clinic. Common networking includes Transmission Control Protocol/Internet Protocol (TCP/IP), but Windows networking is also used. Configuring the network under Windows or one of the varieties of UNIX (LINUX, Solaris, HPUX, etc.) is often required. The next software layer includes Windows or UNIX file‐sharing such as SaMBa (Server Message Block), as well as File Transfer Protocol (FTP) and DICOM communication protocols. In particular, DICOM is becoming a vital requirement; many vendors have embraced this standard not just for images, but also for the transfer of various patient medical information such as radiotherapy treatment plans.

Understanding the relationship between the hardware and the operating system of the computers is vital to ensuring that the machines work as designed. Much of the calibration data for the current generation of medical devices are stored on the hard drives of the various computers that form the systems. These hard drives must be maintained and backed up at a reasonable frequency to ensure a hard drive failure has minimal impact on the operation of the machine. In addition, changes in the operating system settings, such as virtual memory or video bit depth, can greatly affect the usability of the machines. Maintenance or error logs are also generally stored within deep nested directories on the hard drives; finding and interpreting these logs often requires special training and documentation.

Security is also a requirement in these systems, not only for patient safety and confidentiality but also to maintain maximum uptime, given the increased likelihood of having the entire PC‐based system inoperable for several hours due to a virus outbreak in the hospital WANs. Vendors have transitioned from forbidding non‐provided software on their systems to requiring that each hospital provide antivirus software. A balance must be struck between configuring the software so that it does not interfere with the routine operations of the medical device, and providing adequate protection. The field engineer, working with IT staff, is often responsible for implementing these security solutions.

## VI. DETERMINING IT RESOURCES

A clear vision for the goals of the department is necessary when planning for growth and keeping up with technological advancements. The IT training that medical physicists, field service engineers, and dedicated RO IT professionals receive must be sufficient to serve the needs of the RO department. Continuing education is necessary to remain current in a rapidly changing field. This training should also equip them to evaluate a department's IT resource needs, although most of the training still happens “on the job.”

The RO department must recognize the role of the physicist in IT applications and consider the additional hours when performing full‐time employee (FTE) calculations for hiring medical physicists, as such duties are not included in the Abt report.^(21)^ RO leaders (e.g. department chair, chief physicist) should inform and educate IT staff and administration about the IT responsibilities of medical physicists. In some hospitals and clinics, IT personnel are unaware of the existence of the medical physicist and their role in RO IT. Working relationships between IT staff and medical physicists should be established as soon as possible, preferably early in the planning stages for new facilities.

The creation of a robust IT infrastructure requires input from the medical physicists, since they have the best understanding of the nature of the data and its flow through clinical processes. A clear methodology should be followed for these needs assessments, since this analysis must take place prior to the installation of new imaging, planning, documentation (e.g. paperless department), and treatment delivery systems. No recommendation can provide a single one‐size‐fits‐all list. Rather, the following questions need to be addressed for each IT related system: What is needed for archive and backup? Who manages it? How many FTEs are required? What skill set is required to perform the work? Does this require real‐time service? How does this affect the current network topology? How does this change the amount of work required to maintain other systems that interact with this one?

For example, if a clinic decides to acquire a linac upgrade for a localization cone beam CT (CBCT), the number of images and the required storage volume must be estimated, along with the man‐hours required to maintain and troubleshoot the system. The distribution of the images for review at the physicians’ workstations may require a change to the network, depending on the architecture needed by the manufacturer of the CBCT system. It may also require a change to the storage space on the review workstations. If the system fails while localizing a patient (e.g. a full disk prevents the images from being saved), real‐time solutions will be required and IT staff who are familiar with the equipment must be immediately available. The IT man‐hours for this system may need to be divided according to the level of RO‐specific knowledge. This will also depend on the clinic's model for IT staffing.

There are a number of other details that are not included in the previous example, and the analysis of one system has to be taken in the context of the other systems in the department. The complexity increases with the number of systems, and accurate estimates of man‐hours, computing hardware, and networking infrastructure may be difficult to achieve. Hence, the estimates can vary among individuals who determine these resource needs. A resource guideline would be beneficial and a number of articles on the Web describe how to determine IT resource needs,^(22)^ but they all seem to focus on standard computing systems (PCs, networks, mainframes) and do not address the special needs of radiation oncology. There appears to be no publication addressing this issue.

While this white paper does not intend to solve this lack of information with detailed tables of data storage volumes and man‐hours, a few suggestions for creating needs assessment guidelines can be offered. This involves a certain level of abstraction of the types of details given in the CBCT example. CBCT imaging could be characterized as a service (IGRT), or as a high storage volume computing device. These views can lead to a “cafeteria plan” model or a “patient count” model.

In the case of a cafeteria plan model, the various services that are offered in RO can be presented like a menu in terms of the IT man‐hours required for support, the archive storage size, the number of network nodes, and the number of CPUs involved for each specific computing task. These resource lists or “menus” can be scaled according to the number of people (computer users) providing the service. A clinic could create its list of needs by selecting the services it offers from the menu. The menu could be created by surveying well run RO departments, analyzing their services and the corresponding established IT resources. In such a model, one would also have to take into account that some services will share resources, and these redundancies need to be removed from the clinic's final list of needs.

For a patient count model, one would survey efficiently run departments and count the number of computers, imaging, planning, and treatment devices, networking nodes, Internet protocol addresses, backup and storage systems, number of patients, and IT FTEs. The IT resources would then be mapped against the number of patients. A clinic could then determine its IT needs based on the number of patients. This would be a simpler way to perform the analysis. The difficulty with this model, however, is that there may be some treatment techniques that are resource‐intensive — using up more resources, on average, to treat a single patient. In this case, the model would underestimate the needs of such a clinic.

Perhaps a hybrid of the two models would work best, using the number of patients as a baseline and selecting from a menu of resource‐intensive services. There may be other means of performing such a needs analysis that one could borrow from other industries. These ideas need further consideration, and perhaps these concepts could be the subject of an AAPM task group. What is important here is that there is recognition of the complexity of the task, and that guidelines would be helpful in determining whether a resource needs list is accurate. The medical physicist must be involved in the analysis, and obtaining a guideline is only the first step. A more detailed analysis will still be required to provide thorough needs assessments, and guidance on the performance of such analysis would be beneficial to the medical physics community.

Once the needs of a department have been determined, budgets should be prepared with clear explanations of the purpose of each item, and the department responsible for the item should be identified. Budgets should be clearly delineated and follow the schemes described in the three separate tiers. Hospital administrators must be alerted to the danger of grouping similar items together in the name of efficiency. Items differ considerably in terms of purpose and responsible personnel, and should be budgeted accordingly. For example, generic storage space may fall under a general IT budget, and might be easily cut from the budget as a low priority item. However, if its purpose as a critical component in the implementation of data intensive technology (such as IGRT and 4DRT) is considered, it should be included in the budget for the acquisition of the new technology. Administrative decisions that shuffle budget items should be made in consultation with the medical physicist.

Finally, RO is a dynamic field with ever‐improving technology. Plans to deal with the expansion of the department should be part of the IT resource needs assessment. Physicists, in consultation with IT staff, should select appropriate IT technologies that accommodate growth. Flexible, scalable solutions should be researched. As these may come from third party sources, their integration with vendor proprietary solutions must be carefully considered.

## VII. CONCLUSIONS

The ideas discussed in this white paper are the result of issues raised at meetings of the AAPM WGIT, which falls under the AAPM Quality Assurance and Outcome Improvement Subcommittee. The proper management of IT resources is necessary to ensure patient data integrity, plan quality assurance, and availability of real‐time critical patient care, and to maximize the potential for improved treatment outcomes. Improving IT‐dependent clinical processes by managing IT resources with a patient‐centered mindset involves the participation of the medical physicist in patient‐related IT decisions. The medical physicist should collaborate with hospital or clinic IT staff, dedicated RO IT staff, equipment service engineers, and vendors. A clear understanding of RO IT related tasks, resources, and responsibilities should be developed, and the training requisite to these roles should be acquired. In this era of increasing RO IT demands, the importance of having an IT professional, well‐trained in RO and dedicated to the clinic, can not be understated. The inclusion of the medical physicist in all RO IT‐related decisions is essential and should be acknowledged by all key IT decision makers in the hospital.
